# Pathologic complete response after preoperative anti-HER2 therapy correlates with alterations in PTEN, FOXO, phosphorylated Stat5, and autophagy protein signaling

**DOI:** 10.1186/1756-0500-6-507

**Published:** 2013-12-05

**Authors:** Frankie Ann Holmes, Virginia Espina, Lance A Liotta, Yasir M Nagarwala, Michael Danso, Kristi J McIntyre, Cynthia R C Osborne, Thomas Anderson, Lea Krekow, Joanne L Blum, John Pippen, Allison Florance, Janine Mahoney, Joyce A O’Shaughnessy

**Affiliations:** 1 Texas Oncology-Memorial Hermann Memorial City, US Oncology Research, 925 Gessner Road #550, Houston, TX 77024-2546, USA; 2 George Mason University, Manassas, VA, USA; 3 GlaxoSmithKline, Collegeville, PA, USA; 4 Virginia Oncology Associates, US Oncology Research, Norfolk, VA, USA; 5 Texas Oncology-Dallas Presbyterian Hospital, US Oncology Research, Dallas, TX, USA; 6 Texas Oncology-Baylor-Charles A Sammons Cancer Center, US Oncology Research, Dallas, TX, USA; 7 Texas Oncology-Bedford, US Oncology Research, Bedford, TX, USA; 8 GlaxoSmithKline, Research Triangle Park, NC, USA

**Keywords:** Invasive breast cancer, Trastuzumab, Lapatinib, Autophagy, Protein signaling

## Abstract

**Background:**

To define protein molecular characteristics of tumor cells prior to, and immediately following, preoperative human epidermal growth factor receptor 2 (HER2)-targeted therapy that correlate with pathologic complete response (pCR) or non response (no pCR) to preoperative HER2-directed therapy and chemotherapy.

**Methods:**

This open-label, phase II study randomized patients with HER2-positive stage II or III invasive breast cancer to trastuzumab, lapatinib, or both, 2 weeks prior to and during chemotherapy with FEC75 for 4 courses; then paclitaxel 80 mg/m^2^ weekly for 12 courses, then surgery. Core needle biopsies were collected at baseline and after 2 weeks of anti-HER2 therapy prior to chemotherapy. Data were correlated with pCR, defined as absence of invasive tumor in breast and lymph nodes.

**Results:**

Of 100 enrolled patients, the analysis population included those who had surgery and received ≥75% chemotherapy (78% [n = 78]). pCRs by arm are: trastuzumab (n = 26), 54% [n = 14]; lapatinib (n = 29), 45% [n = 13]; trastuzumab plus lapatinib (n = 23), 74% [n = 17]). Paired biopsy specimens were available for 49 patients (63%). Tumor cells of patients with pCR in the trastuzumab or lapatinib treatment arms showed nonphosphorylated FOXO, phosphorylated Stat5, and sparse signal-transduction protein network crosstalk representing different patterns of connections with PI3K and autophagy proteins compared with no pCR.

**Conclusion:**

In this exploratory study, pCR with preoperative anti-HER2 therapy and chemotherapy correlated with the levels and phosphorylation status of specific baseline signal pathway proteins in tumor cells. These data may provide candidate biomarkers to stratify initial treatment and potential combination therapies for future study. Tissue preservation technology introduced here makes this procedure widely feasible.

**Trial registration:**

ClinicalTrials.gov: NCT00524303

## Background

Despite treatment with an effective human epidermal growth factor receptor 2 (HER2)-targeted agent and chemotherapy, approximately 15% of early-stage breast cancer patients relapse [1,2]. Furthermore, the pathologic complete response (pCR) rate to preoperative anti-HER2-targeted therapy and chemotherapy is only 40% to 60% [3]. The HER2 pathway, activated upon HER2 pairing with a partner protein, may be highly complex depending on the partner, the configuration of downstream interconnections, and the state of alternative pathways. This molecular complexity provides multiple mechanisms with which the tumor may evade a therapy targeted to a single pathway [4,5]. Trastuzumab binds within domain IV of the extracellular domain of the HER2 homodimer, but HER2 may also heterodimerize with other HER (ErbB) receptors and non-HER2 partners [4,5]. An effective treatment must target both extant pathways and those activated after treatment. Active pathways are identified by determining the phosphorylation state of pathway proteins, which can be measured by reverse phase protein microarray technology [6].

The objective of this study was to measure the activity state of signal pathway proteins linked to HER2 signaling. Part one evaluated the effect of combining anti-HER2 therapy with chemotherapy (totaling 26 weeks), measured by the pCR rate after definitive surgery. Part two identified the molecular networks involved in HER2 signaling at baseline and how they were altered after two weeks of anti-HER2 treatment alone. Serial biopsies were performed to obtain proteomic data and other molecular biomarker data. Clinical response and safety were also assessed. Part three examined a new, room-temperature, fixation solution.

## Methods

### Study design and patients

This open-label, phase II study (LPT109096; NCT00524303) randomized patients with HER2-positive stage II or III invasive breast cancer to trastuzumab, lapatinib, or both.

Eligibility criteria for patients were: ≥18 years of age; the presence of untreated, biopsy-proven, HER2-positive (immunohistochemistry [IHC] 3+ or fluorescence in situ hybridization [FISH] ratio >2.2), American Joint Commission on Cancer stage II or III invasive breast cancer; Eastern Cooperative Oncology Group (ECOG) performance status of 0 or 1; left ventricular ejection fraction ≥50% and within the institutional range of normal by echocardiogram or multi-gated acquisition scan; the ability to swallow and retain oral medication and to provide written informed consent. Adverse events (AEs) were graded according to National Cancer Institute Common Toxicity Criteria for AEs v3.0 [7].

All data were verified by US Oncology Research and GlaxoSmithKline (US Oncology 05-074, GlaxoSmithKline LPT109096, registration NCT00524303). This study was developed by the Breast Committee of US Oncology Research with GlaxoSmithKline and, in accordance with the precepts of the Helsinki Declaration, was approved by the US Oncology Research central institutional review board, Houston, TX, and clinically performed by US Oncology Research.

### Randomization and masking

**Figure 1:**
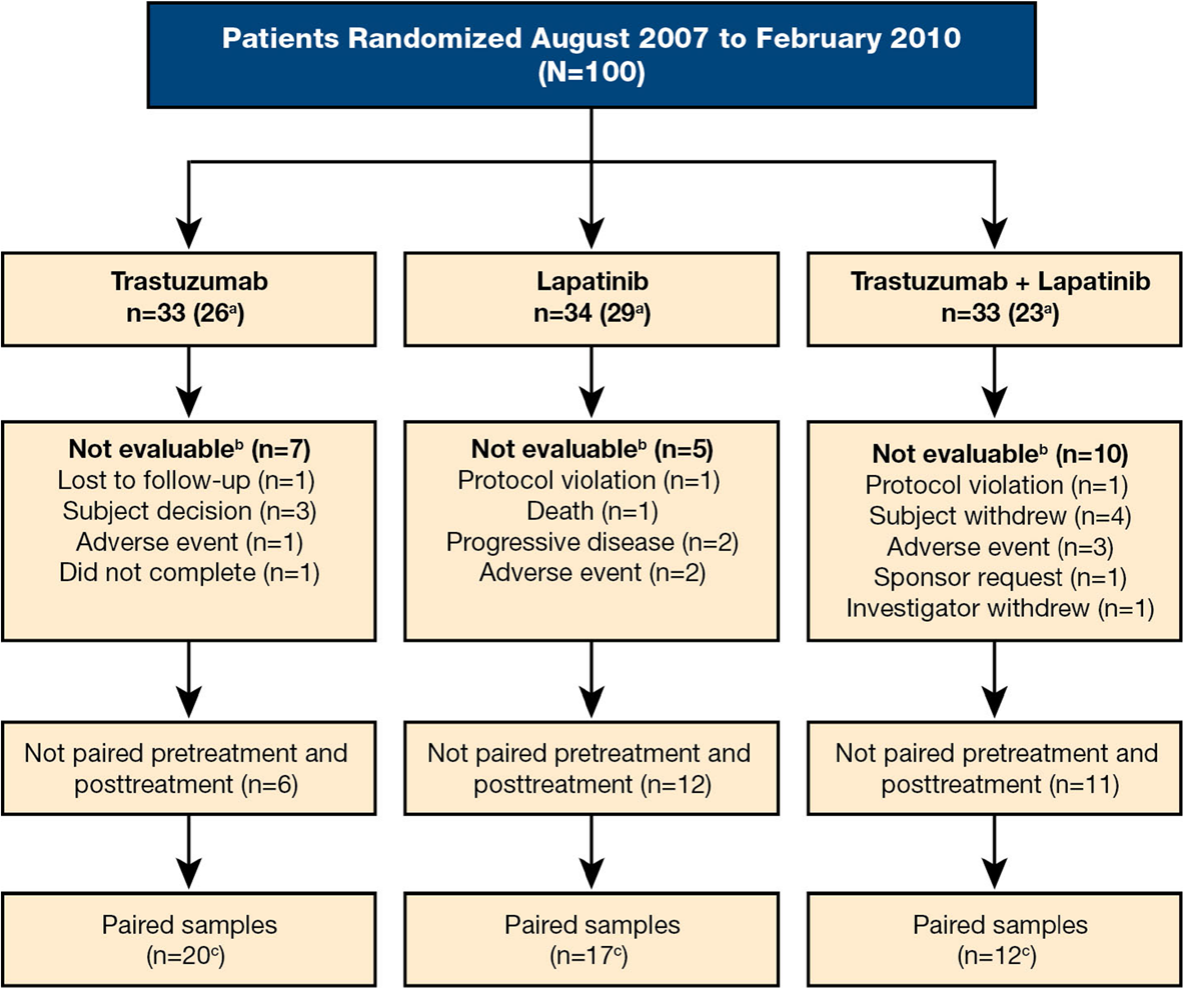
**CONSORT diagram. **^a^Patients who had surgery; ^b^Patients with inevaluable tumor responses; ^c^Patients who had surgery and had paired biopsies for evaluation.

In this open-label study, patients were randomly assigned sequentially using a permutated block design (block size of 6) to receive one of three anti-HER2 therapies: trastuzumab, lapatinib, or the combination (Figure 1). No stratification factors were applied.

### Procedures

Patients received anti-HER2 therapy for 14 days prior to and during chemotherapy and continued until surgery: trastuzumab 4 mg/kg loading dose, then 2 mg/kg weekly; lapatinib 1500 mg daily, reduced to 1250 mg to reduce diarrhea after a safety review of the first 45 patients enrolled (protocol amendment 3, approved April 15, 2009); or trastuzumab given as above with lapatinib 1000 mg daily, reduced to 750 mg daily during the 2-week run-in period and FEC, then increased to 1000 mg during paclitaxel (protocol amendment 3, as above). Chemotherapy was FEC75 intravenously once every 21 days for four treatments, followed by paclitaxel 80 mg/m^2^ intravenously for 12 weeks. Lapatinib was supplied by the sponsor, GlaxoSmithKline. FEC75 includes 5-fluorouracil 500 mg/m^2^, epirubicin 75 mg/m^2^, cyclophosphamide 500 mg/m^2^. Postsurgical treatment was not specified.

### Specimen collection and analysis

Core breast biopsies in the surgeon’s office were collected at baseline and after the first 14 days of anti-HER2 treatment, prior to chemotherapy: 4 cores at each time point. One core from each time point was fixed in a protein/phosphoprotein preservative developed by George Mason University, Manassas, VA [8]. Frozen tissue sections were prepared for laser capture microdissection of breast tumor and/or stroma with analysis by reverse phase protein microarray (Additional file 1). The protein endpoints evaluated are listed in Additional file 2: Table S1. Proteomics analysis was performed by LAL and VE.

### Statistical analysis

The sample size was based on feasibility, estimation of the effect size, and to allow a sufficient number of samples for biomarker evaluation. This study was not powered to provide inference testing for the primary endpoint. Based on a planned total sample size of 99 subjects (33 per regimen), this study had at least 47% power to detect an increased response rate in the comparator, lapatinib, or combination arm compared with the control arm, trastuzumab monotherapy, using a two-sided Fisher exact test at the 5% significance level if the estimated pCR rates in the comparator arms (lapatinib and the combination) compared with the control arm were 75% and 50%, respectively.

All randomized patients comprised the intention-to-treat (ITT) population, regardless of whether they had received treatment. The ITT-evaluable (ITT-E) population consisted of ITT patients with evaluable tumor responses who had received ≥75% of the planned FEC75 and paclitaxel and had surgery. The ITT-E was the primary analysis population for the pCR endpoint. The safety population was all randomized patients who received at least one dose of study treatment.

pCR was estimated for each of the three treatment arms for the ITT-E (primary endpoint) and ITT (sensitivity analysis) populations. Exact 95% confidence intervals (CIs) for the pCR response rates in each treatment arm were calculated. Further exploratory summaries by the subgroups of hormone receptor (HR)-positive (defined as estrogen receptor [ER]-positive and/or progesterone receptor [PR]-positive) and HR-negative (defined as ER-negative and PR-negative) were also examined.

Overall response rate (ORR) was calculated from the review of best response of confirmed partial response and CR only using Response Evaluation Criteria in Solid Tumors 1.0.[9] Subjects with unknown or missing responses were treated as non-responders. Estimates and Fisher exact 95% CIs for the ORR in each treatment group were calculated.

All biomarker analyses were conducted on the ITT-E population. Wilcoxon-rank sum was used to calculate group differences if the data were not normally distributed. Student t-test was used if the data were normally distributed. P values <0.05 were considered significant; no adjustment for multiple comparisons were made. Mean centered data normalization was performed using Z-score transformation. Spearman rho (SR) nonparametric correlations between pairs of endpoints were calculated.

SR correlation statistics were used to determine which protein endpoints were interacting, evidenced by coordinated changes, in each treatment group, ie, either both increased or decreased or both consistently changing in opposite directions, after treatment. The interacting proteins in the network using this standard approach were correlated (p < 0.001) over a large range of the examined network proteins. Ratios of pairs of interacting proteins (documented to show statistically significant SR correlation) in the signal pathway network showed significant differences associated with pCR (Table 1). This suggests activation or suppression of these pathways before or after treatment.

**Table 1:** Protein signal pathway correlates of breast pCR

**Analyte**	**Treatment arm (Baseline/Day 14)**	**Reverse phase protein microarray results**			**Interpretation**
		**pCR Breast Yes**	**pCR**	**p Value <0.05**	
			**Breast ** **No**		
EGFR Tyr1068	Lapatinib arm Baseline	Mean:	Mean:	p = 0.02^c^	Phosphorylation of EGFR at Tyrosine 1068 occurs during receptor activation, leading to downstream activation of growth and pro-survival pathway proteins.
		-0.66	-0.01		
		(n = 6)	(n = 11)		
				
FOXO1/03A Ratios	All treatment arms Baseline				FOXO is a transcription factor that functions as a tumor suppressor. FOXO triggers cell cycle arrest and apoptosis after it enters the nucleus. Phosphorylation of FOXO (pFOXO) causes export from the nucleus and inactivates its tumor suppressor function. Trastuzumab inhibits HER2+ cells by reactivation of FOXO1A [15]
pPTEN:pFOXO		Mean ratio: 2.79	Mean ratio: 0.04	p = 0.01^b^	
		(n = 27)	(n = 22)		
PI3K:pFOXO		Mean ratio: 3.23 (n = 27)	Mean ratio	p = 0.039	
			-0.2 (n = 22)		
pStat5 Tyr694	Trastuzumab arm day 14	Mean:	Mean:	p = 0.017^b^	Stat5 is a transcription factor. After phosphorylation by JAK2, pStat5 translocates to the nucleus to initiate transcription of e-cadherin, which promotes homotypic adherence between breast cells and basement membrane, i.e., normal behavior. Absent nuclear pStat5 is a negative prognostic factor [16,17]. Increased pStat indicates transcription functionality in our pCR subjects.
		1.14	-0.49		
		(n = 11)	(n = 9)		
Protein linkage correlation analysis^a^	All treatment arms Baseline				Autophagy is a normal cellular process to promote cell survival under stress (hypoxia, nutrient deprivation, loss of adhesion). Autophagy allows the cell to “self eat” to generate ATP from cellular contents and thereby survive in times of stress, starvation, hypoxia, or chemotherapy. Autophagy is regulated by a network of cell signaling proteins, including mTOR, Beclin (upstream markers), Bcl2, LC3B (downstream markers), Atg5. Cells from patients classified as pCR No appear to engage autophagy to survive in the presence of treatment with trastuzumab, lapatinib or the combination [18].
LC3B-Beclin			LC3B-Beclin 1 SR = 0.88	p < 0.001	
LC3B-MMP14			LC3B-MMP14	p < 0.001	
			SR = 0.83		
LC3B-Her2		LC3B-HER2		p < 0.001	
		SR^a^ = 0.88			
LC3B-Stat5 Tyr694		LC3B-pStat5		p < 0.001	
		SR = 0.84			

^a^Nonparametric Spearman’s rho (SR) correlation coefficient; ^b^Wilcoxon rank sum; ^c^Two sample t-test.

## Results

Between August 13, 2007 and October 15, 2010, 100 patients at 17 clinical centers were enrolled. The ITT-E population consisted of 78 patients and, of these, 49 had paired specimens for molecular evaluation (Figure 1). Reasons for nonevaluability included no tumor in the specimen, insufficient protein, or poor reproducibility between replicates. Overall, most baseline characteristics were balanced across the treatment arms (Table 2); however, there was an imbalance in HR status, with fewer HR-negative patients in the combination arm. Clinical data cut-off for these analyses was December 17, 2010 and occurred when the last patient reached the time of surgery.

**Table 2:** Baseline patient and tumor characteristics

	**Trastuzumab (n = 33)**	**Lapatinib (n = 34)**	**Trastuzumab + Lapatinib (n = 33)**	**Total (N = 100)**
**Demographics**				
**Age (years)**				
Mean (standard deviation)	51.1 (10.90)	50.8 (8.76)	49.2 (10.47)	50.4 (10.01)
Median (range)	54.0 (21-67)	52.0 (25-67)	50.0 (28-66)	51.5 (21-67)
<65 years, n (%)	31 (94)	32 (94)	30 (91)	93 (93)
≥65 years, n (%)	2 (6)	2 (6)	3 (9)	7 (7)
**Menopausal status, n (%)**				
Postmenopausal	19 (58)	19 (56)	16 (48)	54 (54)
Sterile	5 (15)	5 (15)	6 (18)	16 (16)
Potential to bear children	9 (27)	10 (29)	11 (33)	30 (30)
**Race, n (%)**				
African American	8 (24)	1 (3)	2 (6)	11 (11)
White/Caucasian	25 (76)	27 (79)	29 (88)	81 (81)
Other	0	6 (18)	2 (6)	8 (8)
**Disease characteristics**				
**Histology, n (%)**				
Adenocarcinoma	3 (9)	6 (18)	1 (3)	10 (10)
Medullary	0	0	1 (3)	1 (1)
Lobular invasive	0	1 (3)	2 (6)	3 (3)
Infiltrating ductal NOS	26 (79)	23 (68)	22 (67)	71 (71)
Other	4 (12)	4 (12)	7 (21)	15 (15)
**Grade, n (%)**				
Not assessed	3 (9)	3 (9)	3 (9)	9 (9)
Well differentiated	0	3 (9)	1 (3)	4 (4)
Moderately	10 (30)	7 (21)	12 (36)	29 (29)
Poorly	20 (61)	21 (62)	17 (52)	58 (58)
**Hormone receptor status, n (%)**				
Positive (ER + and/or PR+)	15 (45)	14 (41)	20 (61)	49 (49)
Negative (ER- and PR-)	18 (55)	20 (59)	13 (39)	51 (51)
**HER2-IHC status, n (%)**				
0	2 (6)	0	1 (3)	3 (3)
1+	0	0	1 (3)	1 (1)
2+	4 (12)	3 (9)	0	7 (7)
3+	22 (67)	26 (76)	26 (79)	74 (74)
Unknown	5 (15)	5 (15)	5 (15)	5 (15)
**HER2-FISH status, n (%)**				
Positive	27 (82)	24 (71)	23 (70)	74 (74)
Negative	0	1 (3)	0	1 (1)
Unknown	6 (18)	9 (26)	10 (30)	25 (25)
**Tumor size, n (%)**				
T2	22 (67)	12 (35)	22 (67)	56 (56)
T3	8 (24)	11 (32)	6 (18)	25 (25)
T4	3 (9)	8 (24)	5 (15)	16 (16)
Tx	0	3 (9)	0	3 (3)
**TNM stage: regional nodes, n (%)**				
N0	18 (55)	11 (32)	13 (39)	42 (42)
N1	12 (36)	16 (47)	14 (42)	42 (42)
N2	3 (9)	7 (21)	6 (18)	16 (16)

*Abbreviations:ER* estrogen receptor, *FISH* fluorescence in situ hybridization, *HER2* human epidermal growth factor receptor 2, *IHC* immunohistochemistry, *NOS* not otherwise specified, *PR* progesterone receptor, *TNM* tumor, node, metastasis.

**Figure 2:**
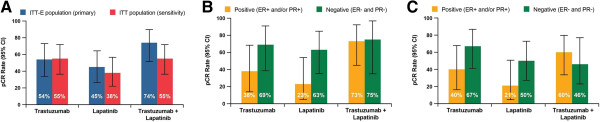
Rate of pCR for (A) ITT-E and ITT population (B) by hormone receptor status of primary in ITT-E population (C) by hormone receptor status in ITT population.

The pCR rate and 95% CIs for the primary ITT-E and ITT populations are shown in Figure 2. In the ITT-E population, the combination arm had a higher pCR rate (74%) compared with the monotherapy arms (trastuzumab and lapatinib, 54% and 45%, respectively). The exploratory analysis of pCR rate for HR status was higher in HR-negative patients than in the HR-positive patients in the monotherapy arms (Figure 2B and C). However, in contrast with other studies, there was no difference in the pCR rate by HR status for the combination arm.

The ORR rates were similar in both the analysis populations (% [95% CI] for trastuzumab, lapatinib, and combination arms, respectively): ITT-E 62 (40.6, 79.8), 76 (56.5, 87.7), 70 (47.1, 86.8); ITT, 61 (42.1, 77.1), 68 (49.5, 82.6), 61 (42.1, 77.1).

Biomarker findings that correlated with pCR in breast in the 49 patients with matched baseline and post-treatment samples are summarized in Table 1. None of the individual baseline protein analytes were significantly different across or between any treatment arms, confirming a lack of bias in pre-existing protein networks. Individual endpoints as well as ratios between two endpoints were evaluated. When we evaluated each treatment arm for individual analyte endpoints, the mean expression levels of pEGFR Tyr1068, at baseline for the lapatinib arm were significantly higher in patients with pCR (n = 11) compared with no pCR (n = 6; p < 0.02) (Table 1). In the trastuzumab arm, the level of pStat5 Y694 post-treatment (day 14) was elevated in the pCR cohort (p < 0.017). When protein analyte ratios were compared with pCR for all treatment arms, the baseline ratios of pPTEN to pFOXO (p < 0.01) and PI3K to pFOXO (p < 0.039) were correlated with pCR (Table 1).

**Figure 3:**
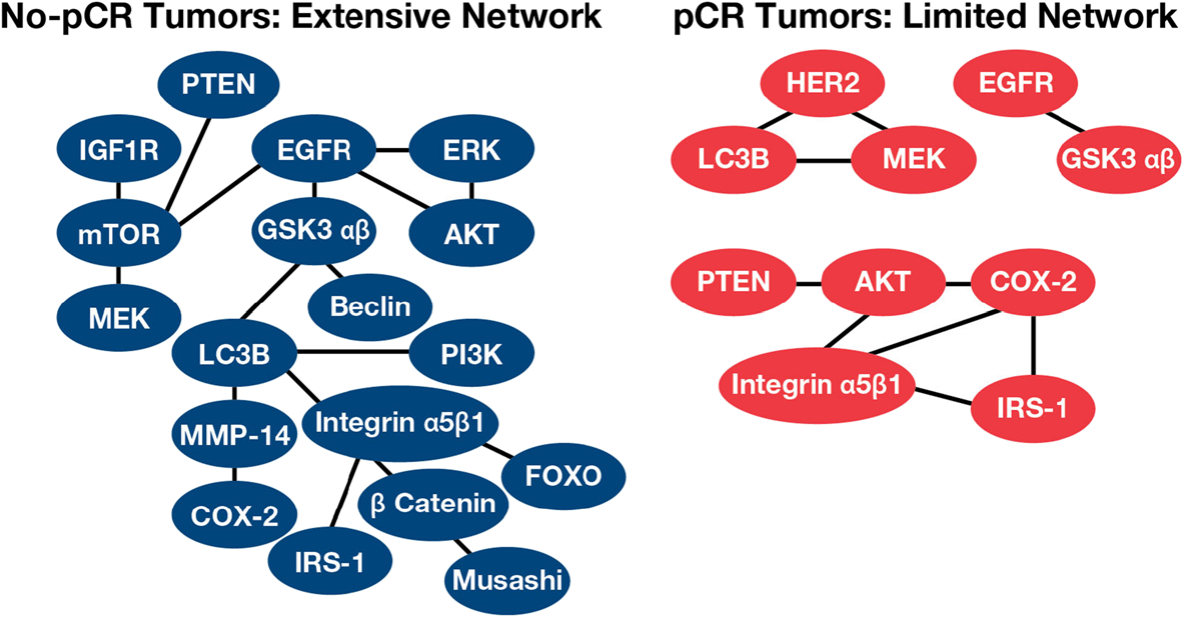
Protein signal pathway interconnections in baseline biopsies.

Two markers of autophagy were correlated with pCR: Beclin1, an initiation marker, and LC3B, an autophagosome marker. Tumors with pCR showed a linkage of baseline LC3B with either HER2 or Stat5 (SR, p < 0.001); whereas, tumors without pCR showed linkage of LC3B with Beclin1 and MMP-14 (SR, p < 0.001). Data from the three treatment arms were combined to determine whether there was a SR protein endpoint linkage association with pCR. In the baseline breast tumor biopsy specimens, there were fewer proteins with significant correlations (22 proteins; p ≤ 0.05) compared with tumors that did not achieve pCR (33 proteins; p ≤ 0.05) as shown in Figure 3.

The safety and tolerability profile of trastuzumab and lapatinib administered with FEC and paclitaxel in this patient population was generally consistent with the known profiles of each individual agent and the chemotherapies administered. No new safety signals were identified. A similar number of patients reported AEs (Table 3 and Additional file 3: Table S2) and serious AEs (SAEs; Additional file 4: Table S3) in the three treatment arms. No SAEs were reported by more than 3 (9%) patients in any treatment group. No fatal AEs were reported on study or within 30 days of last dose of study treatment.

**Table 3:** Summary of all AEs in at least 20% of subjects in any treatment arm (safety population)

	**Arm 1 trastuzumab N = 32**^**a**^	**Arm 2 lapatinib N = 34**	**Arm 3 trastuzumab + Lapatinib N = 31**^**a**^
**Any AE, n (%)**	**31 (97)**	**34 (100)**	**31 (100)**
Diarrhea	17 (53)	29 (85)	31 (100)
Nausea	26 (81)	26 (76)	27 (87)
Rash	14 (44)	28 (82)	26 (84)
Fatigue	22 (69)	24 (71)	24 (77)
Alopecia	21 (66)	23 (68)	18 (58)
Neutropenia	15 (47)	12 (35)	16 (52)
Neuropathy peripheral	15 (47)	19 (56)	14 (45)
Anemia	10 (31)	12 (35)	12 (39)
Hypokalaemia	2 (6)	10 (29)	11 (35)
Epistaxis	7 (22)	10 (29)	10 (32)
Dysgeusia	4 (13)	10 (29)	9 (29)
Myalgia	4 (13)	10 (29)	9 (29)
Pyrexia	3 (9)	8 (24)	9 (29)
Vomiting	7 (22)	15 (44)	9 (29)
Mucosal inflammation	6 (19)	7 (21)	8 (26)
Nail disorder	4 (13)	10 (29)	8 (26)
Cough	8 (25)	3 (9)	7 (23)
Headache	8 (25)	6 (18)	7 (23)
Decreased appetite	5 (16)	7 (21)	7 (23)
Dyspepsia	6 (19)	5 (15)	7 (23)
Dyspnea	4 (13)	6 (18)	7 (23)
Edema peripheral	5 (16)	10 (29)	7 (23)
Stomatitis	5 (16)	7 (21)	7 (23)
Arthralgia	5 (16)	11 (32)	6 (19)
Constipation	12 (38)	8 (24)	6 (19)
Pruritus	3 (9)	7 (21)	6 (19)

*Abbreviation:AE* adverse event.

^a^1 patient in the trastuzumab arm and 2 patients in the trastuzumab + lapatinib arms did not receive therapy and therefore were not included in the safety population.

## Discussion

This small clinical discovery trial of preoperative HER2-positive breast cancer confirmed previous reports that dual-targeted anti-HER2 therapy was more effective at inducing pCR than single-agent therapy [10-12]. The safety profile was consistent with known profiles of the individual agents. There are four important aspects of this study. One, the study verifies a method to conduct extensive proteomic analysis of signal pathways containing the drug target before and after treatment with HER2-directed therapies, before administration of any chemotherapy. Two, samples were collected for RNA, stem-cell analysis, and DNA deep sequencing to be correlated with proteomic data in subsequent reports. Three, this study was performed in a community network, in practicing oncologists’ offices, where the majority of patients usually receive treatment. Four, this study confirmed the effectiveness of preserving phosphoproteins in tissue samples without freezing using a new one-step preservation chemistry [8].

Proteins, and their post-translational modifications, functionally mediate the pathways activated or amplified by genetic abberrations. Although phosphoproteins are valued molecular indicators, accurate proteomic analysis is difficult because they rapidly degrade after tissue devascularization by biopsy or excision [13]. Preserving phosphoproteins has previously required cumbersome techniques [8]. The combination of rapid molecular preservation, laser capture microdissection of the cancer cells, and highly sensitive proteomic microarray technology [8] permitted a large number of proteins and pathways to be evaluated that have not been reported, or are less well known in relation to anti-HER2 therapy. The correlation of the expression of these proteins and the activation of these pathways with pCR suggest molecular vulnerabilities that may provide targets for future individualized treatment trials (see Additional file 5).

Among all the proteins and phosphoproteins measured, only a small subset showed correlations with pCR. These included phosphorylated proteins associated with ErbB1/EGFR, PTEN, FOXO, and Stat5, as well as non-phosphorylated proteins associated with autophagy (LC3B, Beclin1) and extracellular matrix proteolysis (MMP14) (Table 1). Phosphorylation of EGFR at Tyrosine 1068, which correlated with no pCR in the lapatinib arm, occurs during receptor activation, leading to downstream activation of growth and pro-survival pathway proteins that may override suppression by lapatinib; although, these differences were not significant when all baseline samples (n = 65) were analyzed.

FOXO, a transcription factor, functions as a tumor suppressor by initiating cell-cycle arrest and apoptosis after entering the nucleus. Phosphorylation of FOXO causes export from the nucleus, which inactivates the tumor-suppressor function. Trastuzumab inhibits HER2-positive cells by reactivation of FOXO1A [14]. Thus, FOXO1A and pFOXO levels reflect the activity status of the HER2/PI3K/AKT network. We found that, in all treatment arms, pCR tumors had low pFOXO. Thus, a potential strategy for patients with high pFOXO or low pPTEN at baseline may be the addition of a PI3K pathway inhibitor.

Stat5, signal transducer and activator of transcription, is a transcription factor. After phosphorylation by JAK2, pStat5 translocates to the nucleus to initiate e-cadherin transcription, which promotes homotypic adherence between breast cells and basement membrane, ie, normal behavior [15]. Absent nuclear pStat5 is a negative prognostic factor in early-stage breast cancer and predicted increased risk of antiestrogen failure [16]. Studies in chronic lymphocytic leukemia (CLL) cells have shown that treatment with interleukin-21 promotes Stat5 phosphorylation in natural killer cells which enhanced antibody-dependent cellular cytotoxicity (ADCC) against rituximab-coated CLL cells in vitro [17]. Similarly, combined use of anti-ErbB monoclonal antibodies and erlotinib enhanced ADCC in wild-type erlotinib sensitive non-small-cell lung cancer cell lines [18]. Our finding of pStat5 in this study relates to an intracellular signaling protein influenced in its function by phosphorylation; nevertheless, we cannot exclude an indirect effect through ADCC. In our study, tumors that showed higher levels of pStat5 after 14 days of trastuzumab (i.e., normal transcription functionality) had pCR.

Autophagy is a normal cellular process to promote cell survival under stress, i.e., hypoxia, nutrient deprivation, loss of adhesion. Autophagy enables the cell to “self eat” to generate ATP from cellular contents and, thereby, survive during stress or chemotherapy. Autophagy is regulated by a network of cell-signaling proteins including mTOR, Beclin1, Bcl2, LC3B, and Atg5 [19,20]. The no-pCR tumors had active autophagy. Another aspect of autophagy included correlation with MMP-14, which allows a cell to detach from the basement membrane and extracellular matrix, and metastasize, i.e., to survive loss of adhesion. The no-pCR tumors had coordinately increased MMP-14 and LC3B. In cell models of autophagy-induced trastuzumab resistance, RNAi-induced knock-down of LC3B followed by sub-therapeutic doses of trastuzumab caused synergistic growth inhibition. Vazquez-Martin et al. noted that this observation has two implications: 1) hyperactivation of basal autophagy was a survival mechanism in trastuzumab-refractory cells; and 2) inhibitors of autophagy, such as chloroquine, may reverse or prevent trastuzumab resistance [19]. Clinical trials evaluating the worth of autophagy inhibitors are ongoing (ClinicalTrials.gov: NCT01292408; NCT 01009437; NCT01023477).

Network analysis of protein-protein interactions among all the measured proteins in all treatment arms showed that the number and extent of collaborating signal pathways were greater in the no-pCR compared with the pCR tumors. Complete proteomic network analysis of the tumors before and after therapy will be reported separately. These highly networked tumors may engage alternative pathways to survive treatment, thus pretreatment knowledge of these interconnections might allow preemptive therapy of de novo resistance or before acquired resistance.

These study findings are constrained by the small sample size. The protocol specified that patients with paired biopsy specimens would comprise the biomarker-evaluable population. Only 49 (63%) in the ITT-E population had biopsies that contained sufficient invasive cancer and met the required quality control standards for protein assays. The main limitation imposed by the small paired biopsy sample size is the inability to evaluate and compare baseline and day 14 predictive phosphoprotein patterns in each treatment arm.

The small study population may also account for some of the differences between these results and those of trials summarized by von Minckwitz et al., particularly response by HR status [3,10-12,21]. Consistent with other published studies, we found higher pCR rates in the monotherapy arms in HR-negative patients [3,10]. The similar pCR rates in the HR-positive and HR-negative group in the combination trastuzumab/lapatinib cohorts differs from other study findings and could be explained by the wide CIs around the pCR rates due to the small sample size. It is interesting to consider why HR status influences response to HER2-targeted therapies. Xia et al. suggest that one mechanism of acquired resistance to lapatinib in ER-positive/HER2-positive BT474 cells is through de-repression of FOXO3A and enhanced expression of caveolin-1, both of which increase ER-mediated transcription, growth, and survival [22]. In Xia’s model, adding fulvestrant or depleting estrogen from the media reversed the resistance [22].

Nonetheless, von Minckwitz observes pCR is not predictive of disease-free survival in HER2-positive tumors that are HR-positive compared with HR-negative [21]. Thus, while improvement in the pCR rate in this subset has obvious clinical value, e.g., potentially less radical surgery, improvement in the prognostic value of pCR for disease-free survival as well as improvement in the rate of pCR, will require evaluating and targeting multiple pathways in addition to ER and HER2. These data offer potential additional candidate treatment targets and markers to stratify therapy, including FOX03A, HER3, autophagy, and PI3 kinase mutation and/or activation status.

## Conclusions

The findings from this study, if corroborated in larger trials, may identify HER2-positive patients at diagnosis with a high probability of pCR after preoperative anti-HER2 therapy plus chemotherapy, i.e., tumors with high ratios of pPTEN/pFOXO1A and PI3K/pFOXO1A and lack of autophagy (coordinate expression of LC3B with Beclin1 or MMP14). Patients whose cancers have a high degree of PI3K signaling or active autophagy at diagnosis may be candidates for preoperative therapy trials focused on inhibiting these pathways in addition to HER2.

## Abbreviations

AEs: Adverse events; ADCC: Antibody-dependent cellular cytotoxicity; ATP: Adenosine triphosphate; CI: Confidence interval; CLL: Chronic lymphocytic leukemia; DNA: Deoxyribonucleic acid; ECOG: Eastern Cooperative Oncology Group; ER: Estrogen receptor; FEC 5: Fluorouracil, epirubicin and cyclophosphamide FEC; FISH: Fluorescence in situ hybridization; FOXO: Forkhead box 0; HER2: Human epidermal growth factor receptor 2; HR: Hormone receptor; IHC: Immunohistochemistry; ITT: Intention-to-treat; JAK2: Janus kinase 2; MMP: Matrix metalloproteinase; mTOR: Mammalian target of rapamycin; ORR: Overall response rate; pCR: Pathologic complete response; pEGFR Tyr 1068: Phosphorylation of epidermal growth factor receptor at Tyrosine 1068; PR: Progesterone receptor; PTEN: Phosphatase and tensin homologue; RNA: Ribonucleic acid; SAE: Serious adverse event; SR: Spearman rho; TNM: Tumor, node, metastasis.

## Competing interests

This study and editorial support was funded by GlaxoSmithKline. GlaxoSmithKline provided lapatinib, funding, data management, and data analysis and collaborated with US Oncology Research in regulatory and safety monitoring and reporting of the study.

FAH previously served on the speakers’ bureau for Genentech. VE and LAL have an issued patent for phosphoprotein preservative chemistry; in addition, LAL has received honoraria from Theranostics Health. AF, and JM are employed by, or hold leadership positions at, GlaxoSmithKline; they also own stock in GlaxoSmithKline. YMN is a previous employee of GlaxoSmithKline and owns Stock in GlaxoSmithKline. MD, KJM, CRCO, TA, LK, JLB, and JP disclosed no potential conflict of interest. JAO’S is a consultant for, or serves in an advisory role at, GlaxoSmithKline; she also received honoraria from GlaxoSmithKline.

## Authors’ contributions

FAH participated in the design of the study, coordination with investigators and sponsor, analysis, wrote the initial draft and updated subsequent drafts, enrolled and treated patients on the study, and coordinated tissue acquisition at her site. VE and LL prepared the tissue and performed proteomics analysis and statistical analysis of that data. JAOS conceived the study, and participated in its design, coordination, and analysis, and enrolled patients on the study. AF performed statistical analysis of clinical data. YN and JM were the medical monitor and associate who coordinated clinical and biomarker studies and manuscript preparation. MD, KJM, CRCO, TA, LK, JLB, JP enrolled and treated patients on the trial and coordinated tissue acquisition at their respective sites. The corresponding author and the biomarker authors had access to the analyzed data as well as final responsibility for the decision to submit for publication. All authors read and approved the final manuscript.

## Supplementary Material

Additional file 1 Supplementary Information. Click here for file

Additional file 2: Table S1 Protein endpoints analyzed by reverse phase protein microarray. Click here for file

Additional file 3: Table S2 Summary of grade 3 and grade 4 AEs reported in at least 5% of subjects in any treatment arm (safety population). Click here for file

Additional file 4: Table S3 Summary of all SAEs (safety population). Click here for file

Additional file 5 Protein pathway networks analyzed. Click here for file
