# Supplementary material for: Pathologic complete response after preoperative anti-HER2 therapy correlates with alterations in PTEN, FOXO, phosphorylated Stat5, and autophagy protein signaling
Source: BMC Res Notes. 2013 Dec 5;6:507. doi: 10.1186/1756-0500-6-507 (PMC3915616; doi:10.1186/1756-0500-6-507)
Supplement: Additional file 1 — Supplementary Information. [file 1756-0500-6-507-S1.doc]

**Supplementary Information**

**Specimen Preparation**

Core needle biopsy tissue was removed from the George Mason University fixative solution,[8] embedded in Optimal Cutting Temperature compound, and frozen on dry ice. Cryosections were cut on plain glass slides at 8-μm thickness and stored at –80^o^C prior to microdissection.

**Laser Capture Microdissection**

Frozen tissue sections were fixed in 70% ethanol, stained with Mayer’s hematoxylin, dehydrated in graded alcohols (70%, 95%, 100%), and cleared in xylene. Tumor cells and/or stromal cells were microdissected using an Arcturus PixCell IIe (Invitrogen, Life Technologies, Grand Island, NY). Microdissected cells were stored at –80^o^C prior to reverse phase protein microarray printing.

**Reverse Phase Protein Microarray Construction/Staining**

Microdissected cells were subjected to lysis with a 10% (v/v) solution of Tris(2-carboxyethyl) phosphine (Pierce, Rockford, IL) or a 2.5% solution of 2-mercaptioethanol (Sigma) in Tissue Protein Extraction Reagent (T-PER™, Pierce)/ Tris-glycine 2X SDS buffer (Invitrogen). Cell lysates were stored at –80^o^C prior to microarray construction. Cellular lysates were printed on glass backed nitrocellulose array slides (FAST Slides, Whatman, Florham Park, NJ) using an Aushon 2470 arrayer (Aushon BioSystems, Burlington, MA) equipped with 350-μm pins. Each array consisted of microdissected tumor samples. The before/after pair for each patient were printed on the same array for most specimens, which were analyzed in four batches. Samples were printed in duplicate in 4-point or 2-point dilution curves. Immunostaining was performed on a Dako Autostainer per manufacturer’s instructions (CSA kit, Dako, Carpinteria, CA). Each slide was incubated with a single primary antibody at room temperature for 30 minutes. Reverse phase protein microarray analysis of phosphorylated/total protein endpoints included 47 cell-signaling proteins, 31 of which were analyzed in common across all four array sets (Additional file 2). The protein biomarker analytes comprised the known and purported mechanisms of HER2-therapy response and resistance. Antibodies were validated by western blotting.[14, 20–22] The negative control slide was incubated with antibody diluent as a substitute for the primary antibody. Secondary antibody was goat anti-rabbit IgG H+L (1:7,500) (Vector Labs, Burlingame, CA) or rabbit antimouse IgG (1:10) (Dako). Subsequent signal detection was amplified via horseradish peroxidase mediated biotinyl tyramide deposition with chromogenic detection (Diaminobenzidine) per manufacturer’s instructions (Dako). Array slides were scanned at 600dpi on a flatbed scanner (UMAX PowerLook) and saved in a tiff format (Adobe Photoshop). Spot intensity was analyzed using ImageQuant ver 5.2 (GE Healthcare).

Total protein per microarray spot was determined with a Sypro Ruby protein stain (Invitrogen/Molecular Probes) per manufacturer’s directions and imaged with a CCD camera (NovaRay, Alpha Innotech, San Leandro, CA).

**Data Analysis and Reporting**

Data compilation was performed with George Mason University RPMA template ver 6.0, an Excel Macro tool for background correction and normalization. The mean local area background of each spot was used to assess spot intensity values greater than 2 S.D. above background. Replicate values were averaged. If the CV between replicates was >20%, the spot was flagged and a value of “CV too high” was reported. Signal intensity from the secondary antibody alone (negative control) slide was subtracted from the signal intensity of the primary antibody slide. Each patient analyte sample was normalized to the corresponding Beta actin value.

Local area background: signal due to nitrocellulose substratum in the vicinity of each array spot.

Normalized: spot intensity value that has been corrected for background signal and divided by it's corresponding normalizer endpoint value. For example: (ERK signal minus ERK background)/Beta Actin.

Flagged spot: any spot that does not meet our evaluable criteria. Spots were flagged for CV>20% between technical replicates, artifacts on the array, poor spot morphology, or negative spot intensity values.

Quality Control of the Reverse Phase Protein Microarrays

Total protein was analyzed as a quality control marker for each microarray spot. Total protein per spot was quantified as a means to assess overall sample and printing quality. Low total protein levels indicated inadequate sample quality or quantity.

A431, A431+EGF, and BT474 whole cell lysates were printed on each array as quality control samples. Commercially available cell lysates (A431 and A431+EGF) were printed on each array as qualitative assessments of printing and staining processes, i.e. to ensure that protein was deposited on each array and the protein detection reagents (staining) were performing as expected (positive staining with differential expression when probed with various primary/secondary antibody combinations).
