# Supplementary material for: Pathologic complete response after preoperative anti-HER2 therapy correlates with alterations in PTEN, FOXO, phosphorylated Stat5, and autophagy protein signaling
Source: BMC Res Notes. 2013 Dec 5;6:507. doi: 10.1186/1756-0500-6-507 (PMC3915616; doi:10.1186/1756-0500-6-507)
Supplement: Additional file 2: Table S1 — Protein endpoints analyzed by reverse phase protein microarray. [file 1756-0500-6-507-S2.doc]

**Supplemental Table 1**. Protein endpoints analyzed by reverse phase protein microarray.

| **Primary Protein Targets** |  | **Secondary Protein Targets** |  | **Additional Targets Based on Pathophysiology and Preliminary Data** |  | **Common Endpoints Analyzed on All 4 Array Sets** |
| --- | --- | --- | --- | --- | --- | --- |
| AKT |  | CD24 |  | ATG5 |  | **AKT** |
| AKT Ser473 |  | CD44 |  | Beclin 1 |  | **AKT Ser473** |
| AKT Thr308 |  | ERK T202/Y204 |  | CD133 |  | **AKT Thr308** |
| EGFR |  | GSK3 α/ß ser21/9 |  | Cox2 |  | **Beta Catenin Ser33/37/Thr41** |
| EGFR Tyr1068 |  | GSK3 α/ß Y279/Y216 |  | E-cadherin |  | **CD24** |
| EGFR Tyr1148 |  | MEK ser217/221 |  | Fibronectin |  | **CD44** |
| EGFR Tyr1173 |  | mTOR ser2448 |  | Integrin a5b1 |  | **c-erbB2 (total)** |
| EGFR Tyr992 |  | p70S6 Thr389 |  | IRS-1 Ser612 |  | **EGFR** |
| Her2 |  | PI3K |  | LC3B |  | **EGFR Tyr1045** |
| Her2 Tyr1248 |  | Smad2 ser465/467 |  | MMP-9 |  | **EGFR Tyr1068** |
| IGF-1R Tyr1131/1146 |  |  |  | NFKB Ser536 |  | **EGFR Tyr1148** |
| PTEN |  |  |  | p38 MAPK Thr180/Tyr182 |  | **EGFR Tyr1173** |
| PTEN Ser380 |  |  |  | Stat3 Ser727 |  | **EGFR Tyr992** |
| c-erbB2 (total) |  |  |  | Stat3 Tyr705 |  | **ERK Thr202/Tyr204** |
|  |  |  |  | Stat5 Tyr694 |  | **FOX01/03 Thr24/32** |
|  |  |  |  | Timp2 |  | **GSK3 α/ß ser21/9,** |
|  |  |  |  |  |  | **GSK3 α/ß Y279/Y216** |
|  |  |  |  |  |  | **Her2** |
|  |  |  |  |  |  | **Her2 Tyr1248** |
|  |  |  |  |  |  | **Her3 Tyr1289** |
|  |  |  |  |  |  | **IGF1R Tyr1131/1146** |
|  |  |  |  |  |  | **Mek 1/2 Ser217/221** |
|  |  |  |  |  |  | **Met Y1234/1235** |
|  |  |  |  |  |  | **MMP-14** |
|  |  |  |  |  |  | **mTOR Ser2448** |
|  |  |  |  |  |  | **Musashi** |
|  |  |  |  |  |  | **p70S6 Thr389** |
|  |  |  |  |  |  | **PI3K** |
|  |  |  |  |  |  | **PTEN** |
|  |  |  |  |  |  | **PTEN Ser380** |
|  |  |  |  |  |  | **Smad2 Ser465/467** |
