# Supplementary material for: Pathologic complete response after preoperative anti-HER2 therapy correlates with alterations in PTEN, FOXO, phosphorylated Stat5, and autophagy protein signaling
Source: BMC Res Notes. 2013 Dec 5;6:507. doi: 10.1186/1756-0500-6-507 (PMC3915616; doi:10.1186/1756-0500-6-507)
Supplement: Additional file 3: Table S2 — Summary of grade 3 and grade 4 AEs reported in at least 5% of subjects in any treatment arm (safety population). [file 1756-0500-6-507-S3.doc]

| **Supplemental Table 2.** Summary of grade 3 and grade 4 AEs reported in at least 5% of subjects in any treatment arm (safety population) | | | | | | | | | | |
| --- | --- | --- | --- | --- | --- | --- | --- | --- | --- | --- |
|  | | **Trastuzumab** | | | **Lapatinib** | | | **Trastuzumab + Lapatinib** | | |
|  | **All Grades** | | **3** | **4** | **All Grades** | **3** | **4** | **All Grades** | **3** | **4** |
| **Any AE, n (%)** | | **31 (97)** | **10 (31)** | **12 (38)** | **34 (100)** | **12 (35)** | **11 (32)** | **31 (100)** | **9 (29)** | **16 (52)** |
| Diarrhea | | 17 (53) | 1 (3) | 0 | 29 (85) | 8 (24) | 1 (3) | 31 (100) | 14 (45) | 1 (3) |
| Nausea | | 26 (81) | 0 | 0 | 26 (76) | 0 | 0 | 27 (87) | 3 (10) | 1 (3) |
| Rash | | 14 (44) | 0 | 0 | 28 (82) | 2 (6) | 0 | 26 (84) | 5 (16) | 0 |
| Fatigue | | 22 (69) | 0 | 0 | 24 (71) | 1 (3) | 0 | 24 (77) | 2 (6) | 0 |
| Alopecia | | 21 (66) | 2 (6) | 0 | 23 (68) | 0 | 0 | 18 (58) | 2 (6) | 0 |
| Neutropenia | | 15 (47) | 4 (13) | 11 (34) | 12 (35) | 3 (9) | 5 (15) | 16 (52) | 3 (10) | 10 (32) |
| Neuropathy peripheral | | 15 (47) | 0 | 0 | 19 (56) | 2 (6) | 0 | 14 (45) | 1 (3) | 0 |
| Hypokalaemia | | 2 (6) | 1 (3) | 0 | 10 (29) | 1 (3) | 0 | 11 (35) | 3 (10) | 1 (3) |
| Vomiting | | 7 (22) | 0 | 0 | 15 (44) | 0 | 0 | 9 (29) | 3 (10) | 1 (3) |
| Leukopenia | | 3 (9) | 1 (3) | 0 | 4 (12) | 1 (3) | 2 (6) | 5 (16) | 2 (6) | 1 (3) |
| Weight decreased | | 0 | 0 | 0 | 3 (9) | 0 | 0 | 5 (16) | 2 (6) | 0 |
| Febrile neutropenia | | 3 (9) | 3 (9) | 0 | 2 (6) | 1 (3) | 0 | 4 (13) | 3 (10) | 1 (3) |
| Neutrophils decreased | | 2 (6) | 1 (3) | 0 | 4 (12) | 2 (6) | 1 (3) | 3 (10) | 0 | 2 (6) |
| Thrombocytopenia | | 1 (3) | 0 | 0 | 4 (12) | 0 | 0 | 3 (10) | 2 (6) | 0 |
| Syncope | | 1 (3) | 0 | 0 | 3 (9) | 2 (6) | 0 | 2 (6) | 1 (3) | 0 |
| Pulmonary embolism | | 3 (9) | 2 (6) | 0 | 0 | 0 | 0 | 1 (3) | 0 | 1 (3) |
| White blood cell count decreased | | 1 (3) | 0 | 0 | 3 (9) | 2 (6) | 0 | 0 | 0 | 0 |

Abbreviation: AE, adverse event.
