# Supplementary material for: Pathologic complete response after preoperative anti-HER2 therapy correlates with alterations in PTEN, FOXO, phosphorylated Stat5, and autophagy protein signaling
Source: BMC Res Notes. 2013 Dec 5;6:507. doi: 10.1186/1756-0500-6-507 (PMC3915616; doi:10.1186/1756-0500-6-507)
Supplement: Additional file 4: Table S3 — Summary of all SAEs (safety population). [file 1756-0500-6-507-S4.doc]

| **Supplemental Table 3**. Summary of all SAEs (safety population) | | | |
| --- | --- | --- | --- |
|  | **Arm 1**  **Trastuzumab**  **N=32^a^** | **Arm 2**  **Lapatinib**  **N=34** | **Arm 3**  **Trastuzumab + Lapatinib**  **N=31^a^** |
| **Any SAE, n (%)** | **7 (22)** | **7 (21)** | **8 (26)** |
| Diarrhea | 0 | 2 (6) | 2 (6) |
| Febrile neutropenia | 3 (9) | 0 | 2 (6) |
| Neutropenia | 3 (9) | 0 | 1 (3) |
| Pulmonary embolism | 3 (9) | 0 | 1 (3) |
| Dehydration | 0 | 1 (3) | 1 (3) |
| Vomiting | 0 | 1 (3) | 1 (3) |
| Chest discomfort | 0 | 0 | 1 (3) |
| Deep vein thrombosis | 0 | 0 | 1 (3) |
| Dyspnoea | 0 | 0 | 1 (3) |
| Gastroenteritis | 0 | 0 | 1 (3) |
| Liver function test abnormal | 0 | 0 | 1 (3) |
| Nausea | 0 | 0 | 1 (3) |
| Stomatitis | 0 | 0 | 1 (3) |
| Urinary tract infection | 0 | 0 | 1 (3) |
| Pyrexia | 0 | 3 (9) | 0 |
| Cellulitis | 0 | 1 (3) | 0 |
| Diverticulitis | 0 | 1 (3) | 0 |
| Hyponatremia | 0 | 1 (3) | 0 |
| Swelling face | 0 | 1 (3) | 0 |
| Anemia | 1 (3) | 0 | 0 |
| Upper respiratory tract infection | 1 (3) | 0 | 0 |

Abbreviation: SAE, serious adverse event.

^a^1 patient in the trastuzumab arm and 2 patients in the trastuzumab + lapatinib arms did not receive therapy and therefore were not included in the safety population.
