# Supplementary figures and images for: Pathologic complete response after preoperative anti-HER2 therapy correlates with alterations in PTEN, FOXO, phosphorylated Stat5, and autophagy protein signaling
Source: BMC Res Notes. 2013 Dec 5;6:507. doi: 10.1186/1756-0500-6-507 (PMC3915616; doi:10.1186/1756-0500-6-507)

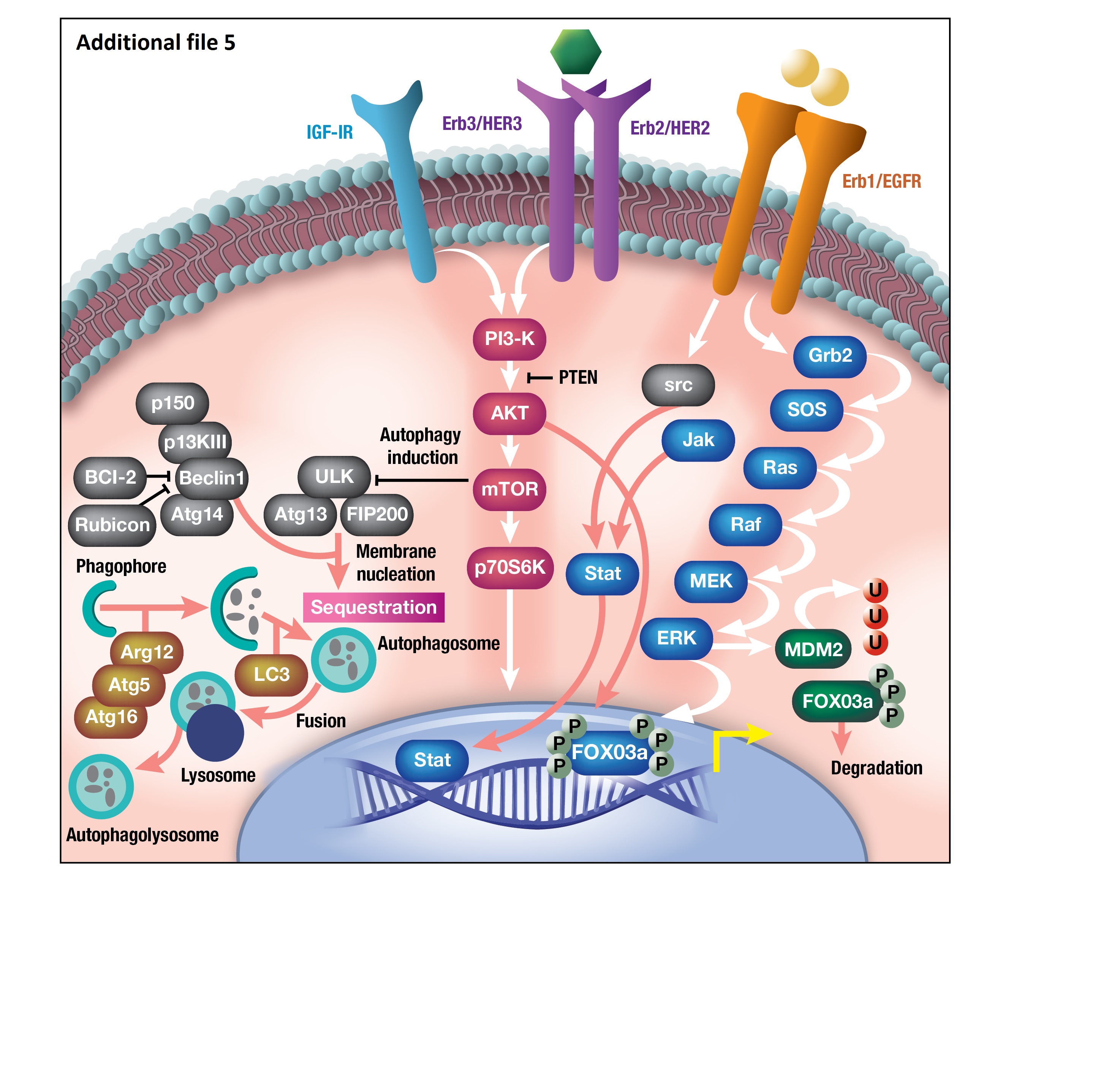

Supplement: Additional file 5 — Protein pathway networks analyzed. [file 1756-0500-6-507-S5.jpeg]
